# The Binding of Aβ42 Peptide Monomers to Sphingomyelin/Cholesterol/Ganglioside Bilayers Assayed by Density Gradient Ultracentrifugation

**DOI:** 10.3390/ijms21051674

**Published:** 2020-02-29

**Authors:** Hasna Ahyayauch, Igor de la Arada, Massimo E. Masserini, José L. R. Arrondo, Félix M. Goñi, Alicia Alonso

**Affiliations:** 1 Instituto Biofisika (CSIC, UPV/EHU) and Departamento de Bioquímica, Universidad del País Vasco, 48080 Bilbao, Spain; ahyayauch@hotmail.com (H.A.); igor.delaarada@ehu.es (I.d.l.A.); gbproarj@ehu.es (J.L.R.A.); felix.goni@ehu.es (F.M.G.); 2 Institut Supérieur des Professions Infirmières et Techniques de Santé, Oujda 60000, Morocco; 3 Neuroendocrinology Unit, Laboratory of Genetics, Neuroendocrinology and Biotechnology, Faculty of Sciences, Ibn Tofail University, 14000 Kénitra, Morocco; 4 School of Medicine and Surgery, University of Milano-Bicocca, 20900 Monza, Italy; massimo.masserini@unimib.it

**Keywords:** Aβ42, beta-amyloid, membrane binding, density gradient ultracentrifugation, ganglioside, sphingomyelin, cholesterol

## Abstract

The binding of Aβ42 peptide monomers to sphingomyelin/cholesterol (1:1 mol ratio) bilayers containing 5 mol% gangliosides (either GM1, or GT1b, or a mixture of brain gangliosides) has been assayed by density gradient ultracentrifugation. This procedure provides a direct method for measuring vesicle-bound peptides after non-bound fraction separation. This centrifugation technique has rarely been used in this context previously. The results show that gangliosides increase by about two-fold the amount of Aβ42 bound to sphingomyelin/cholesterol vesicles. Complementary studies of the same systems using thioflavin T fluorescence, Langmuir monolayers or infrared spectroscopy confirm the ganglioside-dependent increased binding. Furthermore these studies reveal that gangliosides facilitate the aggregation of Aβ42 giving rise to more extended β-sheets. Thus, gangliosides have both a quantitative and a qualitative effect on the binding of Aβ42 to sphingomyelin/cholesterol bilayers.

## 1. Introduction

Alzheimer’s disease (AD) is the most frequent cause of dementia. From its earliest description, the disease has been linked to the presence of plaques on the central nervous system cells. In 1984, Glenner and Wong [1] isolated and sequenced what is now known as the amyloid-β (Aβ) peptide and identified this serum peptide as the major component of amyloid plaques. The Aβ peptide would be derived from the sequential processing of the amyloid precursor protein (APP) by β- and γ-secretases [2,3]. A plethora of theoretical and experimental work has been devoted to the biophysics, biochemistry and pathology of Aβ. It is now accepted that Aβ peptide is released from cells in a soluble form, and progressively undergoes aggregation forming oligomers, multimers, and fibrils, ending with deposition of extracellular plaques [3]. The toxicity of Aβ peptide appears to require conversion of the monomeric form to an aggregated fibrillar species. There is abundant work indicating that cell membranes may play a significant catalytic role in increasing Aβ aggregation rates (see, e.g., [4,5,6,7] for reviews).

Early data [8,9] supported the idea that β-secretase associates with liquid-ordered (Lo) nanodomains in the membrane and that integrity of those raft-like domains is required for β-cleavage of APP to occur. This explains why many studies on Aβ-membrane interaction have been performed with bilayers enriched in sphingomyelin (SM) and cholesterol (Chol), a lipid mixture that exists usually in the Lo phase [10]. Moreover, various reports [11,12] have shown that anionic lipids promote fibril elongation. In our previous study [13] we explored the initial stages of the interaction of Aβ42 in the monomeric form with lipid monolayers and with bilayers composed mainly of SM and Chol, i.e., in the liquid-ordered (Lo) state, in the absence and presence of negatively charged phospholipids. In the absence of negatively charged lipids, the interaction was weak. However, in the presence of phosphatidic acid, or of cardiolipin, an interaction could be detected by different methods, including isothermal calorimetry, thioflavin T (ThT) fluorescence and infrared spectroscopy, as well as molecular dynamics simulations. Low (2.5–5 mol %) concentrations of phosphatidic acid or cardiolipin allowed better interaction than 20 mol % of the same lipids.

For many years gangliosides have been associated to plaque formation [14], perhaps because of their presumed implication in raft structure [15]. Numerous studies have explored model membranes composed of SM, Chol and gangliosides. Selected examples include those based on molecular dynamics [16,17,18], atomic force microscopy (AFM) [19,20], infrared spectroscopy [21], and other biophysical techniques [22,23]. To our knowledge Aβ42 binding to bilayers after separation of the free and lipid-bound forms of the peptide by density gradient centrifugation have rarely been performed. Vesicle-bound peptide floats in the density gradient whereas the non-bound protein/peptide, after aggregation, sediments [24,25]. The centrifugation method is quite unique in providing direct, quantitative equilibrium measurements of binding, in the absence of added probes or chemical modification of the ligand. In the present work we have extended our previous studies to Lo model membranes containing gangliosides, either GM1, or GT1b, or a mixture of brain gangliosides. We have assessed Aβ binding to bilayers using density gradient centrifugation. Our results have been validated using other classical techniques, such as ThT fluorescence, Langmuir monolayers, or IR spectroscopy. Quantitative data of the ganglioside effects on Aβ binding have been determined.

## 2. Results

### 2.1. Binding Assessment through Flotation Assays

The binding of Aβ to LUV was assessed by floating a vesicle + peptide mixture through a sucrose density gradient under centrifugal force. Vesicle-bound peptides float through the lower density layers, while the lipid-free Aβ aggregate and sediment with or below the bottom layer. Four fractions, F1 to F4 from bottom to top, were retrieved from the ultracentrifuge tube. The rhodamine-stained material was visible, under a UV lamp, only in fractions F3 and F4. In addition, some fluorescent material remained adhered to the tube walls (fraction L), that was solubilized in SDS for further quantification. Table 1 shows the percent distribution of Aβ in the various fractions for vesicles consisting of SM/Chol (1:1 mol ratio) or SM/Chol/ganglioside (47.5:47.5:5 mol ratio). In the latter case experiments with a mixture of brain gangliosides, or of pure GM1, or of pure GT1b, were performed. Aβ recovery after the gradient was ≈100% in all cases.

The results show that binding was highest (≈90% in F4) with GM1 or GT1b, somewhat lower with the ganglioside natural mixture (≈84% in F4, with ≈11% non-vesicle-bound), and lowest for the liposomes lacking ganglioside (only ≈41% in F4, with ≈32% non-vesicle-bound Aβ). This provides the most direct evidence to date of the long-proposed positive role of gangliosides in facilitating Aβ binding to lipid bilayers. The role of gangliosides as facilitators was shown, in less direct ways, by different authors [15,17,18,19,20,21]. Amaro et al. [17] had indicated that, under certain conditions, gangliosides inhibited Aβ oligomerization induced by SM. Gobi et al. [26] measured the binding of Aβ to liposomes containing GM1 by discontinuous gradient ultracentrifugation, but only very weak binding (about 0.5% Aβ vs. 40—90 % in our case, Table 1) was detected either with SM/Chol liposomes, or with SM/Chol + GM1 liposomes by those authors with Aβ monomers. The difference in binding might be due to the use of much lower starting lipid:protein ratios by Gobbi et al. than in our case (5:1 vs. 200:1).

### 2.2. Thioflavin T Assays 

**Figure 1:**
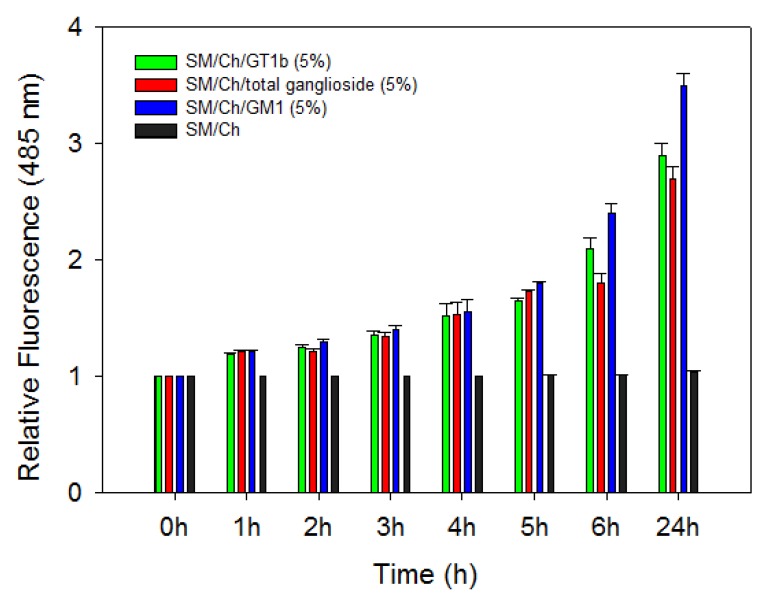
Time course of thioflavin T relative fluorescence emission after addition of Aβ42 to a LUV suspension containing SM/Chol (1:1 mol ratio) ± 5 mol% gangliosides. The data have been normalized to time 0. Average values ± S.E. (*n* = 3).

We used the ThT assay for determination of β-sheet content as this has been often used as a semi-quantitative indication of β-sheet formation by Aβ42. β-sheet formation is associated to the oligomerization process that follows Aβ42 binding to certain lipid bilayers [2,3]. Figure 1 depicts the time-course of ThT fluorescence emission (485 nm) for the first 24 h after liposome-peptide mixing. Fluorescence does not vary for the ganglioside-free SM/Chol vesicles, indicating the absence of β-sheet formation. However, in the presence of 5 mol% gangliosides, an increase in ThT fluorescence is already detected after 1 h, and the effect appears to increase exponentially along the experiment. Note that after 5 h the increase is lower for the total ganglioside than for GM1 or GT1b, in agreement with the binding results in Table 1. The fact that ganglioside-free SM/Chol bilayers bind Aβ (Table 1) but β-sheet does not appear to form (Figure 1), may be due either to a hypothetical need of a certain minimum surface concentration of peptide, that would not be reached in the absence of gangliosides, and/or to a qualitatively different sort of binding that would be induced by Aβ42 binding to a ganglioside molecule. The latter possibility looks more probable, in view of previous observations using a variety of techniques [16,18,27].

### 2.3. Infrared Spectroscopic Studies 

**Figure 2:**
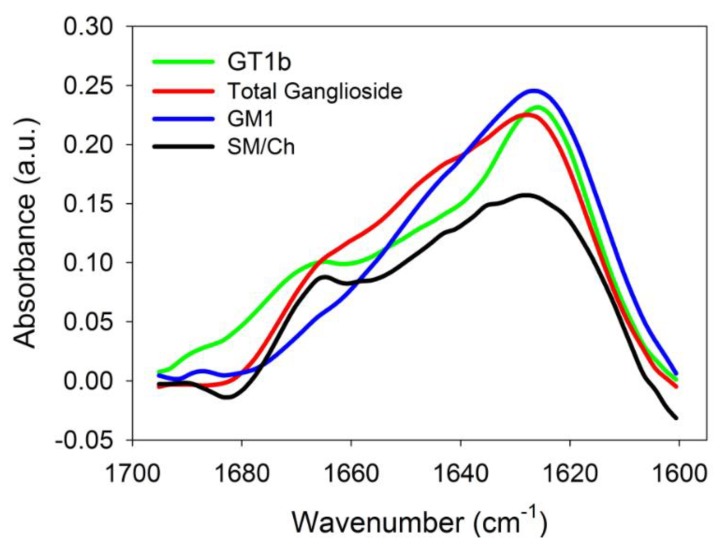
The 1600–1700 cm^−1^ region of the IR spectrum of Aβ42 in mixtures with vesicles of various lipid compositions.

IR spectroscopy is an excellent tool to analyse the secondary structure of proteins in solution [28]. The formation of β-segments upon binding of Aβ42 to bilayers was directly demonstrated by this technique (Figure 2). The spectra were recorded under conditions similar to those in the ThT experiments, i.e., 1:200 peptide/lipid mol ratio. The spectral region corresponding to the amide I band of Aβ42 is shown in the Figure. Control experiments showed that in this spectral region, under our experimental conditions, the lipid signal was contained in the base line, without contributing in a detectable way to the peptide spectra. For the peptide-lipid samples, a predominant β structure was seen in all cases, as shown by the bands centred at ≈1626 cm^−1^ [28]. A shoulder near 1667 cm^−1^, whose contribution was relatively more important for the SM/Chol sample, i.e., without gangliosides, reflected turns or non-periodic structures, that should be more abundant in the free peptides [28]. Correspondingly the intensity of the β-segment signal at ≈1626 cm^−1^ increased in the presence of gangliosides, in agreement with the above results. This confirmed the hypothesis that gangliosides, in addition to facilitating Aβ binding to bilayers, could also enhance the degree of aggregation.

### 2.4. Langmuir Balance Measurements

The surface-active properties of Aβ42 and its monomeric interaction with membrane lipids were tested in a Langmuir balance at the air-water interface, in order to obtain additional information on the ganglioside effects upon Aβ binding. In the absence of lipids, injection of Aβ42 into the aqueous phase caused an increase in surface pressure, the latter reaching equilibrium after ≈1 h. This occurred because Aβ42 is surface active, like many other peptides [29]. The increase in surface pressure was dose-dependent and reached a plateau at ∼10 mN/m, for Aβ concentrations slightly above 1 μM, as described in our previous study [13]. Thus, at these and higher concentrations, the interface was saturated with adsorbed peptide and the peptide partitioned between the interface and the bulk water [29].

**Figure 3:**
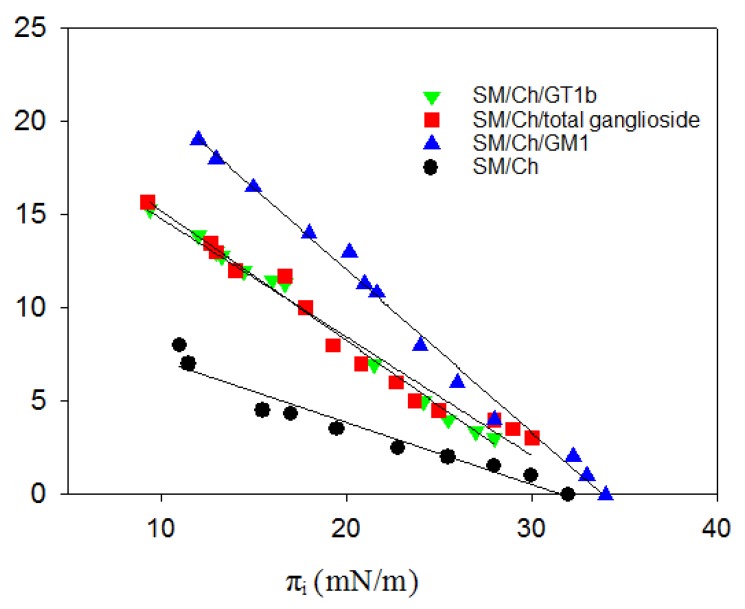
Changes in surface pressure of lipid monolayers, upon insertion of Aβ42 monomers at varying initial pressures (equilibrium values). Average values ± SE (*n* = 3). (Error bars are the same size or smaller than the symbols). Aβ42 stock solution was 50 μM. Aβ42 final concentration in the trough was 1.22 μM.

Aβ insertion into lipid monolayers was assayed in experiments wherein a lipid monolayer was extended at the air-water interface and the peptide was injected into the aqueous subphase. The initial surface pressure of the lipid monolayer was fixed as desired, at values >10 mN/m to avoid simultaneous peptide insertion and peptide adsorption. Aβ insertion into the lipid monolayer at the interface causes a further increase in surface pressure Δπ (Figure 3). For this system, as the initial pressure increased, Δπ decreased (Figure 3) until a point was reached, at 32–34 mN/m, beyond which peptide insertion was no longer possible. Figure 3 includes the results for four different lipid compositions in the monolayer, corresponding to the mixtures used to form the LUV in the above experiments. In the 10–30 mN/m range, peptide insertion became easier in the following order: SM/Chol < SM/Chol/total gangliosides ≈ SM/Chol/GT1b < SM/Chol/GM1. However, the limiting initial pressure for all four lipid compositions was the same, close to 32–34 mN/m. The monolayer data were independent of geometric fluctuations that could be produced by gangliosides in bilayers, and they confirmed that gangliosides facilitate the binding of Aβ42 to membrane lipids.

## 3. Materials and Methods

Aβ42 was generously supplied by Mario Negri Institute (Milan, Italy). ThT was purchased from Sigma (St. Louis, MO). Chol, egg SM, GM1 ganglioside (ovine brain), GT1b (porcine brain) and total ganglioside extract (porcine brain) were from Avanti Polar Lipids (Alabaster, AL, USA).

Aβ42 stock solution was prepared by dissolving the peptide at 1 mg/mL in 1,1,1,3,3,3-hexafluoro-2-propanol to render monomeric Aβ42. The volatile solvent was removed under vacuum in a Speed Vac (ThermoSavant, Holbrook, NY, USA). The predominant monomeric form at least in the first 6 h after preparation was checked by ThT fluorescence [13,30].

Vesicles consisting of SM/Chol (1:1 mol ratio) or SM/Chol/ganglioside (47.5:47.5:5 mol ratio) were used. For liposome preparation, the appropriate lipids were dissolved in chloroform/methanol (2:1, *v*/*v*), and the mixture was evaporated to dryness under a stream of nitrogen. Traces of solvent were removed by evacuating the samples under high vacuum for at least 2 h. The samples were hydrated in 20 mm Tris-HCl, 150 mm NaCl, pH 7.5 (Tris buffer), helping dispersion by stirring with a glass rod. The solution was frozen in liquid nitrogen and thawed 10 times. LUV were prepared by the extrusion method [31], using polycarbonate filters with a pore size of 0.1 μm (Nuclepore, Pleasanton, CA, USA).

For floating assays, rhodamine-stained liposomes were used (0.5 mol % Rho-PE) to facilitate detection. Aβ (15 µM) was incubated with 3 mM liposomes for 2 h at 37 °C in Tris buffer. 100 μL protein/lipid mix was diluted to 200 µL in Hepes buffer containing 2.4 M sucrose (Sigma-Aldrich, S0389). Then, the reaction mix was transferred to a centrifuge tube. The 1.4 M sucrose layer was overlaid with 400 µL Tris buffer containing 0.8 M sucrose and 300 µL of Tris buffer containing 0.5 M sucrose. Sucrose step gradients were centrifuged in a TLA-120.2 rotor (Beckman Coulter, 357656, Brea, CA, US) at 356,160× *g* for 3 h at 4 °C. Four 250-µL fractions (F1 – F4) were pipetted, starting from the bottom. Fractions F3 and F4, containing liposomes as indicated by the rhodamine fluorescence, plus an additional fraction (L) resulting from washing the tube with SDS were analyzed by densitometric analysis of SDS-PAGE Tris-tricine gels stained with Coomassie Blue.

ThT fluorescence increases with β-sheet contents in the peptide. In our case the test was applied to SM:Chol mixtures with or without gangliosides. ThT fluorescence assays were performed essentially as described by Nilsson [30]. Briefly, ThT was prepared in glycine (50 mM, pH 8.2) and filtered (0.22 mm). Stock Aβ42 was added to each vesicle solution to yield incubation mixtures containing 5 μM Aβ with 1:200 peptide to lipid mole ratio at 37 °C. ThT was then added. Following gentle mixing, the fluorescence was recorded in an Aminco-Bowman (Urbana, IL, USA) AB-2 spectrofluorometer (λex = 446 nm, λem = 485 nm). Control samples of pure Aβ and peptide-free vesicles were also prepared and measured as indicated.

For infrared spectroscopy measurements [32], the mixtures of vesicles and Aβ were dialyzed against Tris buffer + 1 mM EDTA at 4 °C overnight. The suspension was then concentrated to 2 mg protein ml^−1^ and next dialyzed against Tris buffer at 4 °C for 6 h in order to remove EDTA and excess salts. Protein aliquots (0.1 mL) were freeze-dried, then resuspended in the appropriate volume of D_2_O-based buffer and the infrared spectra were recorded. Samples contained 80 μM Aβ42, at a 1:200 peptide: lipid mole ratio. The spectra were retrieved in a Bruker Tensor 27 (Bruker OptikGmbH, Ettlingen, Germany) spectrometer equipped with a liquid nitrogen-refrigerated mercury-cadmium-telluride detector. Samples were measured with excavated calcium fluoride BioCell windows (BioTools), and a 25-μm optical path. A total of 143 interferograms min^−1^ were generated at 2 cm^−1^ resolution and averaged over 1 min intervals. Opus 5.0 software from Bruker Optics was used for data acquisition.

Monolayers at the air-water interface in a Langmuir balance were studied at 22 °C as indicated in [29]. In summary lateral pressure experiments were carried out at 22 °C in a µTrough-S system from Kibron (Helsinki, Finland) under constant stirring. The aqueous phase consisted of 1.0 mL Tris buffer. The lipid, about 2 nmol, dissolved in chloroform:methanol (2:1), was gently spread over the surface until the desired initial surface pressure was attained. After allowing for solvent evaporation the Aβ42 peptide was injected with a micropipette through a hole connected to the subphase. Aβ 42 final concentration in the trough was 1.22 μM. The increment in surface pressure versus time was recorded until a stable signal was obtained. The figures show average values of two closely similar independent measurements.

## 4. Discussion

The above data describe the interaction of the Alzheimer-related peptide Aβ42 with lipid monolayers and bilayers composed of SM/Chol (1:1 mol ratio) in the presence and absence of gangliosides. The main conclusion, that is univocally supported by the four experimental techniques used in the study, is that gangliosides facilitate the binding of Aβ42 to the bilayer while modifying the peptide conformation, specifically increasing its β-sheet contents. As a methodological novelty, binding has been measured directly, under equilibrium conditions, and in the absence of chemical probes, using density gradient ultracentrifugation to separate the free from the bound peptide. These results deserve some comment in the light of pertinent data in the literature.

It was suggested that β-secretase, the protease responsible for the cleavage of APP yielding Aβ42, was associated to the so-called rafts, membrane nanodomains in the Lo state [8,9,33,34] and that integrity of those raft domains was essential for β-cleavage of APP to occur. Rafts are supposed to be enriched in sphingolipids and cholesterol, consequently many studies of Aβ42 interaction with membranes, including the present one, have been carried out with lipid mixtures containing SM, gangliosides and Chol. However, the parallelism between the model membrane studies and the situation in cell membranes is not immediate, for at least two reasons. One is that rafts are supposed to be nanodomains [15], i.e., orders of magnitude smaller than the smallest liposomes or supported planar bilayers, and this difference in size is currently understood to have large consequences on the physical properties of the object [35], thus it would be risky to translate directly results from the micro-to the nano-scale. The second source of uncertainty comes from the lack of factual data on the lipid composition of the proposed rafts [15], for which qualitative data are insecure and quantitative data are outright inexistent. Thus the biophysical data obtained with micrometre-sized model membranes must be interpreted with extra precaution.

A related problem arises from the quantitatively very different compositions in model membrane studies. Limiting ourselves to the phospholipid/cholesterol/ganglioside mixtures, ganglioside concentration may vary from 1–2 mol% [17] to 33 mol% [20], while cholesterol is used in the range 10 mol% [27] to 47.5 mol% (in this work). These differences have an obvious influence on the final outcome of the peptide-lipid interaction. Different ganglioside concentrations may either accelerate or inhibit amyloid formation by the pancreatic islet amyloid polypeptide [36] or by Aβ42 (Ahyayauch, H., unpublished results), and this may in turn have pathological consequences [37]. There are also contradictory data on the role of Chol in Aβ42-binding to bilayers, from those that suggest a positive role in Aβ42 binding and aggregation [19,38] to those who propose a negative influence [39] or a dual effect depending on Chol concentration [40]. In general, Chol appears to have a negative effect on Aβ binding and aggregation particularly in the absence of sphingolipids [41,42].

It can be concluded that density gradient centrifugation is a reliable method for the direct measurement of Aβ42 binding to lipid vesicles. This method allows measurement of peptide binding independently of peptide conformational changes. The combined data from the various techniques used in this study demonstrate that gangliosides facilitate binding of Aβ42 to SM/Chol membranes and also enhance the conformational changes leading to β-sheet formation and presumably Aβ42 cluster formation.

## Figures and Tables

**Table 1:** Binding of Aβ42 to sphingomyelin/cholesterol vesicles in the presence or absence of gangliosides: Flotation assay. Percent distribution of peptide in the various gradient fractions: F1, F2, F3, F4 from bottom to top of the centrifuge tube. L, fraction obtained from washing the tube walls with SDS. Average values ± S.E. (n. d. = not detected).

Fractions					
	F1	F2	F3	F4	L
Bilayer composition					
SM/Chol (1:1 mol ratio)	n.d.	n.d.	28 ± 2.7	41 ± 3.3	30 ± 2.2
SM/Chol + 5 mol% total gangliosides	n.d.	n.d.	4.9 ± 0.1	84 ± 3.9	11 ± 1.4
SM/Chol + 5 mol% GM1	n.d.	n.d.	8.2 ± 0.3	91 ± 1.7	n.d.
SM/Chol + 5 mol% GT1b	n.d.	n.d.	10 ± 1.1	89 ± 1.3	n.d.
